# The role of HER2/neu, BCL2, p53 genes and proliferating cell nuclear protein as molecular prognostic parameters in localized prostate carcinoma

**DOI:** 10.1590/S1516-31802004000300009

**Published:** 2004-05-06

**Authors:** Gilvan Neiva Fonseca, Miguel Srougi, Katia Ramos Moreira Leite, Luciano João Nesrallah, Valdemar Ortiz

**Keywords:** Prostatic neoplasms, Genes, Tumor suppressor genes, Oncogenes, Genes, p53, Proliferating cell nuclear antigen, Receptor erbB-2, Neoplasias prostáticas, Receptor erbB-2, Genes p53, Proteína nuclear de proliferação celular, Oncogenes, Genes supressores de tumor

## Abstract

**CONTEXT:**

Prostate cancer is the most frequent solid genitourinary neoplasm in men. Involvement of several genes has been described in the promotion and progression of prostate carcinoma.

**OBJECTIVE:**

To study the expression of the oncogenes HER2/neu and BCL2, tumor suppressor gene p53 and the tumor proliferation rate in 150 radical prostatectomy specimens, in order to define their role as prognostic parameters in localized prostate cancer.

**TYPE OF STUDY:**

Prospective study.

**SETTING:**

Universidade Federal de São Paulo and Hospital Sírio Libanês, Sao Paulo

**PARTICIPANTS:**

One hundred and fifty men who were submitted to radical prostatectomy between August 1997 and August 1998, for localized prostate cancer.

**MAIN MEASUREMENTS:**

All specimens underwent evaluation in their entirety, to determine tumor volume percentage, tumor extent and Gleason score. Immunohistochemistry was performed to determine gene expression using anti- HER2/neu, BCL2 and p53 antibodies, and proliferating cell nuclear antigen. The chi-squared test was used for correlation between gene expression, proliferative activity and histological variables.

**RESULTS:**

Thirty percent of the cases were p53 positive. There was positive correlation between p53 expression and tumor stage. The p53 expression was 22.9% and 42.6% for pT2 and pT3 tumors, respectively (p = 0.01). Expression of HER2/neu, BCL2 and proliferating cell nuclear antigen was identified in 66%, 23% and 43% of patients, respectively. There was no correlation between these three parameters and tumor volume, Gleason score or tumor stage.

**CONCLUSION:**

One-third of prostate adenocarcinomas express p53 protein, and this characteristic is related to tumor stage. HER2/neu is frequently expressed in prostate carcinomas, with no correlation with histological parameters. BCL2 is rarely expressed, and together with proliferative activity has no relationship with prognostic pathological variables in these neoplasms.

## RESUMO

**CONTEXTO:**

Câncer de próstata é a neoplasia geniturinária sólida mais freqüente no homem. Alguns genes foram identificados na iniciação e progressão do carcinoma de próstata.

**OBJETIVO:**

Estudar a expressão dos oncogenes HER2/neu e BCL2, do gene supressor p53, e da taxa de proliferação tumoral em 150 espécimes de prostatectomia radical, para definir o papel prognóstico desses parâmetros no câncer de próstata localizado.

**TIPO DE ESTUDO:**

Estudo prospectivo.

**LOCAL:**

Universidade Federal de São Paulo e Hospital Sírio Libanês, São Paulo.

**PARTICIPANTES:**

Cento e cinqüenta homens foram submetidos a prostatectomia radical entre agosto 1997 e agosto 1998, por câncer de próstata localizado.

**VARIÁVEIS ESTUDADAS:**

Todos os espécimes foram submetidos à avaliação da porcentagem de volume tumoral, da extensão do tumor e da escala de Gleason. Imunohistoquímica foi realizada para determinar a expressão genética dos seguintes anticorpos: anti-HER2/neu, BCL2, p53, e proteína nuclear de proliferação celular. O teste qui-quadrado foi utilizado na correlação entre a expressão genética, a ativi-dade proliferativa e as variáveis histológicas.

**RESULTADOS:**

Trinta por cento dos casos eram p53 positivos. Houve correlação positiva entre a expressão do p53 e o estágio tumoral. A porcentagem de expressão do p53 foi de 22.9% e de 42.6% para tumores pT2 e pT3, respectivamente, (p = 0,01). As expressões de HER2/neu, BCL2 e proteína nuclear de proliferação celular foram identificadas em 66%, 23% e 43% dos pacientes, respectivamente. Não houve correlação entre esses três parâmetros e o volume tumoral, a escala de Gleason ou o estágio da neoplasia.

**CONCLUSÃO:**

Um terço dos adenocarcinomas prostáticos expressam a proteína p53, e essa característica está relacionada ao estágio tumoral. HER2/neu está freqüentemente expressado nos carcinomas de próstata, mas não existe correlação com os parâmetros histológicos. BCL2 e proteína nuclear de proliferação celular raramente estão expressados, não havendo correlação destes com as variáveis de prognóstico patológicos nessa neoplasia.

## INTRODUCTION

Prostate cancer accounts for 41% of all new cancers diagnosed each year in the United States^1^ and is the most frequent solid genitourinary neoplasm in men.^2^ In Brazil, the number of new cases of prostate cancer is now estimated at more than 35,000 per year.^3^ Multiple interactive factors such as hormone levels, diet and environment are related to the developing of prostate carcinoma.^4^

The involvement of several genes has been described in the promotion and progression of prostate carcinoma. HER2/neu is a protooncogene located in chromosome 17q21-22. In prostate adenocarcinomas, the role of HER2/neu is controversial, mainly because of technical problems related to the immunohistochemistry method.^5,6^ Determining of the role of HER2/neu has been a matter of interest since the report of Visakorpi suggesting that hormone-independent tumors switch signaling from the androgen receptor to the HER2/neu receptor.^7^

BCL2 is an oncogene with anti-apoptotic activity. It has been related to androgen-independent prostate adenocarcinomas,^8^ high S-phase and deoxyribonucleic acid (DNA) aneuploidy.^9^ p53 is a tumor suppressor gene located in the chromosome 17p13.1. Its mutation has been described in more than half of human tumors and it codes for a protein with abnormal stability and long half-life that allows its detection via immunohistochemistry using specific antibodies.^10^ p53 expression and mutation has been correlated with advanced disease, loss of tumor differentiation and the transition from androgen-dependent to androgen-independent growth.^11-13^

Proliferating cell nuclear antigen is an auxiliary protein for DNA polymerase-delta that is involved in DNA synthesis and DNA repair. The activity of proliferating cell nuclear antigen has been evaluated in a number of tumors and has been related to prognosis in some of them.^14^ Proliferative activity tends to be low in prostate cancer,^15^ and higher rates have been related to higher tumor stage and disease-specific survival.^16^

The aim of this study was to evaluate tumor proliferation and the expression of the oncogenes HER2/neu, BCL2 and tumor suppressor gene p53 in patients undergoing pros-tatectomy for localized prostate carcinoma, and to define their prognostic roles through correlation with final Gleason score, tumor stage and tumor volume.

## MATERIAL AND METHODS

### Patients and morphological specimen studies.

One hundred and fifty patients with prostate cancer, diagnosed via biopsy and clinically staged as T1c-T2, underwent radical prostatectomy between August 1997 and June 1998 at the Hospital Sírio Libanês, São Paulo, Brazil. The mean patient age was 62 years (range 41-75). The surgical specimens were fixed in 10% buffered formalin for no longer than four hours. The entire surgical margin was stained with India ink, the left and right lobes were separated, 3-mm transverse serial sections were taken from each lobe, and the entire gland was submitted for histological examination. Sections of the bladder neck, prostatic apex, seminal vesicles, and pelvic lymph nodes were also submitted. Between 16 and 20 slides from each gland were examined. The Gleason score was used for histo-logical grading.^17^

The tumor volume was evaluated as described by Humphrey and Vollmer.^18^ A grid, with the tumor area previously sketched out, was placed below the slides. The percentage of tumor on a slide was determined by dividing the number of squares depicting tumor by the number of squares occupied by the whole section. Tumor volume was defined as the mean percentage of tumor in the prostate gland (the percentage of tumor on each slide divided by the number of slides from the prostate gland). Extraprostatic involvement was defined as tumor infiltration of the periprostatic adipose tissue, the neurovascular bundles, or the seminal vesicles. The TNM system was used for tumor staging.^19^

For statistical analysis, the tumors were considered well differentiated when Gleason score was 6 or less, and moderately to poorly differentiated when the Gleason score was 7 to 10. The tumor volume was identified as ≤ 20% or > 20% of the whole gland volume. The disease was considered to be either confined to the gland, or non-confined when it infiltrated the extraprostatic tissue or involved seminal vesicles or iliac lymph nodes.

### Immunohistochemical Analysis

Three-micrometer sections from the paraffin block that best represented the tumor were placed on adhesive-coated slides. In a thermal antigen retrieval process,^20^ the slides were placed in citrate buffer (1 mM, pH 6.0) and heated three times in a domestic microwave oven at high power, for eight minutes each time. The slides were incubated at 4° C overnight, using monoclonal antibodies for HER2/neu (e2-4001+3B5), BCL2 (124), p53 (DO7) and proliferating cell nuclear antigen (PC 10) (Dako, Glostrup, Denmark) at respective dilutions of 1:200, 1:100, 1:50, 1:200 in phosphate-buffered saline solution.

Biotinylated antimouse immunoglobulin G was applied at 1:200 dilution at room temperature for 60 minutes. The slides were rinsed using phosphate-buffered saline solution for 30 minutes, incubated with peroxidase-conjugated streptavidin (streptABC Kit, Dako) at 1:400 dilution in phosphate-buffered saline at room temperature for 45 minutes, and then rinsed again using phosphate-buffered saline for 30 minutes. The color was developed by incubating the slides in 0.06% diaminobenzidine in phosphate-buffered saline for 15 minutes, and the slides were then rinsed in tap water, counterstained using Harris hematoxylin, dehydrated, coverslipped, and viewed under an optical microscope.^21^ Positive and negative controls were performed for each reaction.

For HER2/neu, strong brown staining of the membrane or cytoplasm was considered positive. BCL2 was considered expressed when the cytoplasm turned brown. Both oncogenes were considered to be positive or negative, without gradation. For p53, any number of stained nuclei was considered positive. For proliferating cell nuclear antigen, at least 500 cells were counted, and cells with dark brown nuclear staining were considered positive.

### Statistical Analysis

To test significant relationships among the variables studied, the chi-squared test was used for qualitative variables. p values were considered significant when lower than 0.05. The statistical analysis was performed using the computer program Statistical Package for Social Science (SPSS), version 8 (SPSS, Inc., Chicago, Illinois, USA).

## RESULTS

The mean Gleason score was 6.4, with a range from 4 to 10. The mean and median tumor volumes were, respectively, 23.6% and 21%. Disease was confined to the gland in 95 patients (63.3%) and was staged as pT2. The tumor extended beyond the prostate in 55 patients (36.7%); these cases were staged as pT3. Extra-prostatic tissue was infiltrated in 54 patients (36%) and the seminal vesicles were involved in 18 of the cases (12%). In four patients (2.7%) there was lymph node metastasis.

Ninety-nine (66%) and 35 (23%) of the tumors were positive for HER2/neu and BCL2, respectively. The correlation between the expression of HER2/neu and BCL2 and histological parameters is shown in Table 1. There was no association between HER2/neu or BCL2 expression and tumor volume, Gleason score or tumor stage.

**Table 1:** Correlation between oncogenes (HER2/neu and BCL2), tumor suppressor gene (p53), tumor proliferation rate (proliferating cell nuclear antigen), and specimen pathological variables in prostatic cancer patients, operated in Hospital Sírio Libanês, São Paulo

Parameter	Volume			Gleason score			Tumor extension		
	≤ 20%	> 20%	p	≤ 6	> 7	p	Confined	Non-confined	p
HER2/neu	62	68	0.32	62	71	0.23	70	59	0.19
BCL2	24	22	0.77	19	29	0.13	23	24	0.87
P53	24	36	0.13	25	36	0.12	23	43	0.01
PCNA	37	50	0.09	38	49	0.17	39	52	0.11

*PCNA: proliferating cell nuclear antigen.*

Forty-five tumors (30%) were positive for p53. The correlation between p53 expression and the histological parameters is presented in Table 1. There was a positive relationship between p53 protein expression and tumor stage.

The proliferating cell nuclear antigen presented a mean of 16.7%; the median was 14% and the range was from 1% to 66%. There was no association between cell proliferation and histological parameters (Table 1).

## DISCUSSION

Several studies have sought parameters to predict the outcome for patients with prostate cancer. Certain histological data – Gleason score; the presence and percentage of a tertiary, less differentiated Gleason pattern; tumor volume; and tumor stage – are the best prognosticators of tumor progression.^22-24^

Recent studies have focused on genes related to tumor behavior as efficient predictors of patient outcome. Our study showed p53 expression in 30% of the tumors, which was directly related to tumor stage. In an earlier study, our group was able to find p53 expression in 31% of 118 prostate carcinomas and could prove the association of p53 with tumor stage^13^. Previous reports have described p53 detection in 5% to 79% of prostate cancers.^25,26^ Navone showed p53 expression in 4.4% of localized prostate tumors and in 45% of bone metastasis.^11^ The positive relationship of p53 to tumor grade was very significant in another series, in which p53 was expressed in only 7.5% of well-differentiated prostatic carcinomas and in 80% of poorly differentiated tumors.^27^ Quinn et al. defined p53 expression as tumors presenting clusters of 12 positive cells within a 200-power magnification field and showed p53 expression in 52% of the cases.^12^In this study, p53 expression had strong correlation with relapse and the disease-specific mortality.

The HER2/neu oncogene, located on chromosome 17q21-22, encodes a 185-kDa transmembrane tyrosine kinase growth-factor receptor homologous to the EGFr (E). Since the development of an anti-p185neu antibody (trastuzumab) for the treatment of breast carcinoma, the interest in studying HER2/neu in relation to its expression in different cancers has risen. We were able to detect HER2/neu expression in 66% of our cases, a higher rate than in other studies, with no correlation with the histological parameters. Tissue fixing and processing, and different antibodies used for detection, affect the staining level. The interpretation of the results can also explain some discrepancies seen in the literature.^28^ Despite these controversies, results similar to those seen in our study have been published, in which poor correlation is established between HER2/neu expression and the pathological outcome in prostate cancer.^29,30^

Several studies have reported that BCL2 and proliferation activity are associated with histological prognostic parameters and patient prognosis in prostate cancer.^31-34^ We were unable to prove such correlation, which therefore makes these two parameters unreliable molecular predictors of prostate cancer behavior.

## CONCLUSION

Our study has shown that expression of the HER2/neu oncogene occurs in a significant number of patients with early prostate cancer, thus suggesting its active participation in the first steps of local carcino-genesis. The expression of BCL2, p53 and proliferating cell nuclear antigen is less frequent in such patients, thus indicating their secondary role in the early process of prostate cancer development. The most important finding of our study is that the frequency of p53 protein expression is correlated with more advanced states of prostate cancer. Therefore, patients that have tumors with altered p53 expression should be viewed more carefully, since they are at greater risk of tumor progression and unfavorable outcome.
